# Land-Use Impact on Stand Structure and Fruit Yield of *Tamarindus indica* L. in the Drylands of Southeastern Ethiopia

**DOI:** 10.3390/life11050408

**Published:** 2021-04-30

**Authors:** Gizachew Zeleke, Tatek Dejene, Wubalem Tadesse, Pablo Martín-Pinto

**Affiliations:** 1 Sustainable Forest Management Research Institute, University of Valladolid, Avda. Madrid 44, 34071 Palencia, Spain; gizachewz@eefri.gov.et (G.Z.); pmpinto@pvs.uva.es (P.M.-P.); 2 Ethiopian Environment and Forest Research Institute, P. O. Box 30708, Addis Ababa 1000, Ethiopia; wubalem-tadesse@eefri.gov.et

**Keywords:** bushland, characteristics, dendrometric, farmland, indigenous fruit tree, population status, wild fruit

## Abstract

In this study, we evaluated stand status, dendrometric variables, and fruit production of Tamarind (*Tamarindus indica* L.) trees growing in bushland and farmland-use types in dryland areas of Ethiopia. The vegetation survey was conducted using the point-centered quarter method. The fruit yield of 54 trees was also evaluated. Tree density and fruit production in ha were estimated. There was a significant difference in Tamarind tree density between the two land-use types (*p* = 0.01). The mean fruit yield of farmland trees was significantly higher than that of bushland trees. However, Tamarind has unsustainable structure on farmlands. Differences in the dendrometric characteristics of trees were also observed between the two land-use types. Predictive models were selected for Tamarind fruit yield estimations in both land-use types. Although the majority of farmland trees produced <5000 fruit year^−1^, the selection of Tamarind germplasm in its natural ranges could improve production. Thus, the development of management plans to establish stands that have a more balanced diameter structure and thereby ensure continuity of the population and fruit yields is required in this area, particularly in the farmlands. This baseline information could assist elsewhere in areas that are facing similar challenges for the species due to land-use change.

## 1. Introduction

The wide array of altitudinal gradients and the varying topographic features of Ethiopia have created quite diverse ecological conditions, each with their own characteristic flora and fauna [1]. Approximately 55% of Ethiopian land is arid to semi-arid [2] and, hence, drylands are one of the major agro-ecological zones found in this country [2,3]. In these areas, people depend heavily on natural resources [4,5], and on services provided by ecosystems and agro-ecological systems [2]. Indeed, the majority of the people derive their livelihood from agriculture combined with extensive livestock breeding and the use of a variety of natural products [3]. Among these services, non-timber forest products (NTFPs) play a vital role [6]. In total, 88 valuable species producing NTFPs have been recorded in the dryland forests of Ethiopia [6]. If they are managed wisely, NTFPs can provide a sustainable stream of income and subsistence products while supporting other economic activities via the provision of ecosystem services [7].

*Tamarindus indica* L. (family Fabaceae) is an indigenous NTFP-producing tree species that is found in sub-Saharan Africa [8,9,10]. In Ethiopia, Tamarind are found in *Combretum–Terminalia* and *Acacia–Commiphora* woodlands vegetation types [8]. Tamarind has an ecological and economic significance [11]. Ecologically, it is important because it can grow well under a wide range of soil and climatic conditions and, hence, is found in semiarid areas, low-altitude wooded grassland, savannah, and bushlands [11] and also found along streams and riverbanks [8]. Tamarind can also grow at altitudes ranging from 0 to 1500 m above sea level (m asl) with an annual temperature range of 20–33 °C and annual precipitation range of 350–2700 mm [8]. The extensive root system of the Tamarind tree contributes to its resistance to drought and wind. Thus, Tamarind is potentially suitable for future reforestation or restoration of moisture-deficient arid and semiarid areas [12]. Economically, Tamarind is important because its fruit has a wide range of domestic and industrial uses [13]. In Ethiopia, the fruit is used for food and medicinal purposes by local communities [14]. In addition, rural people sell Tamarind fruit in local markets to generate income to supplement the household economy [15].

Despite the benefits that can be derived from the management and proper conservation of Tamarind trees, the progressive forest land-use change, particularly the conversion of forest lands to agricultural fields of crops in dryland areas of Ethiopia, has imposed pressure on Tamarind trees [6,14]. This land-use change has had a serious impact on the Tamarind tree population, because it depends on natural regeneration [16]. Furthermore, the available information generally lacks adequate quantitative analysis for the development of economic opportunities based on local resources such as Tamarind trees as an alternative to excessive import of exotic products [17]. Although some studies have been undertaken to investigate the fruit, morphological characteristics, and use of Tamarind in different localities in Ethiopia [14,16], the species is still inadequately characterized. Thus, studying how land-use influences the population structure of Tamarind trees might help us to develop effective conservation strategies in accordance with the needs of the local population. In particular, a better understanding of Tamarind dimensional structure could be used as the basis for strong management decisions if this is combined with information about the species spatial distribution [18], patterns of use and harvesting [19,20]. Furthermore, given the contribution of Tamarind fruit to the livelihoods of rural people in Ethiopia, an evaluation of potential yields and important tree parameters that influence fruit production are imperative to enable further improvement and management of this tree.

In this study, we assessed the population patterns and dendrometric characteristics of Tamarind trees growing on farmland and bushland-use types in the Dello Menna district, Bale zone, Southeastern Ethiopia. We hypothesized that land-use type affects the density and population pattern of Tamarind trees in the studied district. In this sense, as this species provides a highly appreciated fruit, the farmers try to conserve some Tamarind trees to cover this demand. Thus, our expectation was to find a higher number of these largest-size Tamarind trees in farmlands when comparing with bushlands due to the lack of competence with other bushes and trees. In addition, we expected to find a gap between regeneration and midterm-size trees, since the seedlings are frequently removed after the rainy period when the farmers prepare the soil for new cultivation. We also hypothesized that the dendrometric characteristics such as crown structure and number of branches of Tamarind trees would vary between the two land uses and, in turn, that such variation would have an impact on the fruit yield of Tamarind trees. Thus, the specific aims of this study were to assess the stand status of *T. indica*, fruit production and dendrometric variables by comparing the farmland and bushland-use types. The information generated from this study provides baseline information that can be used to promote the conservation and sustainable use of Tamarind trees and other valuable wild edible tree species under different land-use types in Ethiopia and could also assist other countries with valuable wild trees that are facing similar issues due to land-use change.

## 2. Materials and Methods

### 2.1. Description of the Study Area

**Figure 1:**
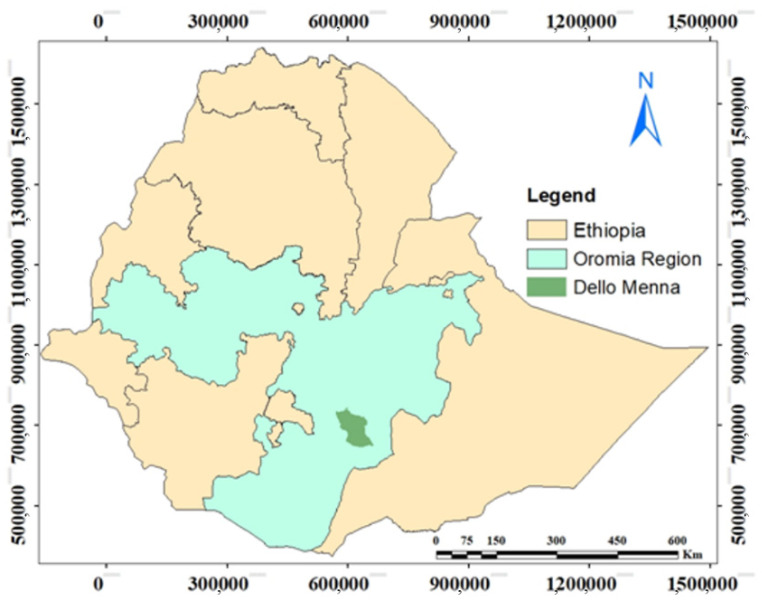
Map showing the location of the study area, the Dello Menna district, in the Oromia region of Southeastern Ethiopia.

The study was conducted in the Dello Menna district (between 5°53′ N and 6°27′ N and between 39°15′ E and 40°38′ E) in the Bale zone of Oromia regional state, Southeastern Ethiopia (Figure 1). Dello Menna is 800 to 2000 m asl and characterized by bimodal rainfall. The main rainy season occurs from early March until the end of June, followed by a shorter rainy season from late September until the end of November. Therefore, there are five dry months in this area: i.e., January, February, July, August, and December. The mean annual rainfall is 986 mm, and the mean annual temperature is 22.3 °C. The average annual rainfall and temperature of the study area during the year (2019) of data collection were 1113.67 mm and 21.26 °C, respectively, indicating that the rainfall amount was good during this period of time. The study area has a plain topography with a few areas of rugged and mountainous terrain. Nitisol is the dominant soil type in this area [21], ranging from a reddish-brown clay toward the higher altitudes and tending to form a reddish-orange sandy soil toward the lower altitudes. Rocky outcrops are prevalent along streams and steeply sloping hills. The inhabitants of the area are primarily Oromo. They practice a mixed farming system involving livestock and subsistence agriculture, which is the main livelihood of the rural community [22]. Coffee, bananas, and papayas are the main perennial cash crops. Teff, sorghum, and maize are the main annual crops cultivated by farmers.

### 2.2. Vegetation Inventory and Data Collection

Woody vegetation characteristics, such as species composition, density, and size structure, were measured by placing transects across the two major land-use types in the study district, namely farmland and bushlands in 2019. For this purpose, we used the point-centered quarter (PCQ) method, a “plot-less” sampling technique, at points along transects across the sampled areas [23]. The transect direction was determined randomly by selecting a bearing from the center of each land-use system, with another transect perpendicular to the first transect (i.e., two cross-cutting transects at 90 degrees). A series of points 100 m apart were systematically located along each transect. A total of 25 and 29 sampling points were used for farmland and bushland-use types, respectively. At every sampling point, four quadrants (90 degrees) were created using the transect line and a line perpendicular to it. The measurement and recording of species in each quadrant was carried out in two stages. Firstly, the *T. indica* that was closest to the sampling point in each of the four quadrants was selected (all sizes were sampled, i.e., seedlings, and mature trees) and then its distance from the central point was recorded. Tree diameters above the basal swell were also measured. Secondly, any woody plant species closest to the sampling point within each of the four quadrants were selected, and the distance from the central point and the tree diameter was recorded. The names of all species registered in the quadrants were noted to determine the species composition.

### 2.3. Fruit Yield Estimation

The productivity of each Tamarind tree in terms of fruit yield was measured using the harvesting method described by Sundriyal and Sundriyal [24]. In total, 27 individual fruit-producing Tamarind trees in each land-use type were randomly selected [25]. The total number of branches and the number of fruit-bearing branches were also recorded during the fruit-harvesting period, as described by Cunningham [25]. To obtain an accurate record of the number of fruit produced per tree, Tamarind fruit were counted when fully matured [26] (i.e., when pods had turned from green to brown) but before the fruit had fallen or been eaten by wild animals. The number of fruit on each terminal branch was determined by either standing on the ground to count fruit hanging down below the canopy, or by climbing the tree to count those in the canopy, or by using a ladder to count those at the top of the crown. Fruit numbers and their weight were recorded for each terminal branch separately and summed for each branch and then the total for each tree obtained by summation. In addition, tree dendrometric variables such as height, diameter at breast height (DBH), crown diameter, and number of branches were recorded [27]. Crown surface area, crown volume, and crown depth were estimated using the measurements obtained for the other dendrometric variables. The DBH was measured using a diameter tape and total height was measured using a Suunto Clinometer. The crown diameter was measured using a cross-method, where the length of the longest spread from edge to edge across the crown and the longest spread perpendicular to the first cross-section through the central mass of the crown were measured. The crown diameter is the average of these two lengths. The number of branches on each tree was also counted in the field.

### 2.4. Data Analysis

To assess the impact of land use on the Tamarind tree species, we compared variables relating to population structure, dendrometric characteristics, and fruit yield. Data analyses were performed using Statistical Package for Social Sciences (SPSS) version 23. Data were log-transformed when needed to achieve the parametric criteria of normality and homoscedasticity necessary for analysis of variance. Differences between the two land-use types for the different variables were evaluated using one-way analysis of variance (ANOVA). The relationship between fruit yield and individual tree attributes was determined through multinomial classification model approach. The models were fitted to the responses to predict the probabilities of the different possible outcomes of fruit yield categories [28]. The analysis used total fruit yield categories as a dependent variable, whereas combinations of dendrometric characteristics were used as explanatory variables. We grouped the response variables as tree size (DBH, height), crown structure (crown diameter, crown depth, crown surface and crown volume) and tree branches (number of branches). For each dependent variable, fruit yield was divided into yield categories (very low, low, high and very high yield) [29]. Then, we combined all the possible combinations, and as a result, we compared the Akaike Information Criterion (AIC) values to select the most suitable model for each studied system. For a given data set, the smaller the AIC, the better the model [30]. Once we obtained the best variable combinations, the model allowed us to predict the probability of the fruit yield for a particular multinomial discrete categorical choice. Coefficients of each model were used to estimate the fruit yield categories of Tamarind trees.

The Crown Surface Area (CSA) estimated using the formula CSA = 2πrh and Crown Volume (CVol) estimated using the formula CVol = 2/3 (πr^2^h), where π = 3.14, r = crown radius and h = the crown depth, which is calculated from the lower side of the crown from tree height. This is now included in the text of the methodology part and the change is highlighted in the text. The crown depth is a parameter used to calculate the crown volume and surface area of individual trees by subtracting the height to the lower side of the crown from tree height.

The population structure of Tamarind trees in both land-use types was assessed by analyzing size class diameters using tree density data collected from PCQ [23,31]. Five diameter classes were used in this assessment: seedlings (recently regenerated); 1 to 10 cm; 11 to 20 cm; 21 to 30 cm; and >30 cm.

- Determination of total distances between trees and sampling points.
- The average distance of the sampled tree from the point, d, was calculated.
- The mean area was determined using the formula mean area = (d)^2^.
- The absolute density of all tree species (trees per ha) was calculated: absolute density = mean area/10,000 m^2^.
- The formulas Fa = Na/n and Fr = (Fa/ΣFai)*100% were used to determine the absolute and relative frequency, respectively, where:
  - Fa is the absolute frequency of the target species;
  - Na is the number of points where at least one individual species occurs;
  - n is the total number of sampling points;
  - Fr is the relative frequency of the target species; and
  - ΣFai is the sum of absolute frequencies of all the species in a transect.
- The relative density of Tamarind was determined by dividing the total number of Tamarind trees by the total number of trees and is expressed as a percentage [23].

## 3. Results

### 3.1. Species Composition, Densities, and Stand Structure

**Figure 2:**
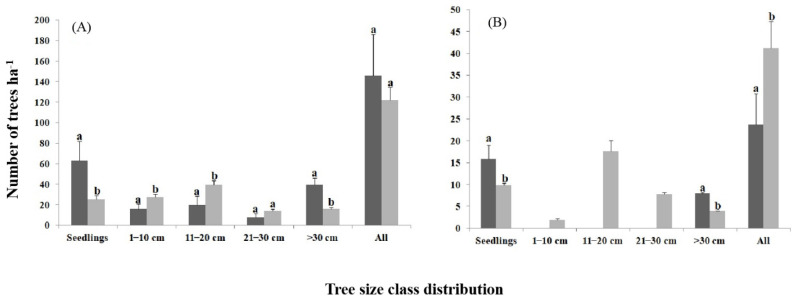
Size class distribution (based on diameter at breast height, DBH) of all tree species (**A**), and of the *Tamarindus indica* tree (**B**) on farmland (dark-gray bars) and in bushland (light-gray) in Dello Menna, Southeastern Ethiopia. Error bars indicate the standard deviation of the mean. Values with the same letter within each size class are not significantly different.

In total, 16 woody tree species belonging to nine families and 12 genera were recorded during the survey of the study area (Table 1). The most prevalent families were the Fabaceae (five species), followed by the Combretaceae (three species), and Malvaceae (two species). These three families represented 53% of the recorded trees, whereas the remaining 47% of families were represented by only a single tree (Table 1). In addition to *T. indica*, *Dobera glabra* was also identified on farmland as an edible fruit tree that was utilized by local communities in the study area. When considering all the trees recorded in each of the land-use types, we found significant differences in the diameter class distribution (*p* < 0.05; Figure 2A). Significantly more trees in the 1–10 cm and 11–20 cm diameter classes were found in bushland than on farmland. However, significantly more regeneration (i.e., seedlings) (*p* = 0.02) and trees with a diameter greater than 30 cm were found on farmland than in bushland (*p* = 0.035; Figure 2A). The total population status of all the trees recorded for the two land-use types is shown in Figure 2A.

Analysis of the *T. indica* population revealed that a significantly greater number of mature Tamarind trees were recorded in bushland than on farmland (F = 10.59, *p* = 0.01; Figure 2B; Table 1). However, a significantly greater number of Tamarind trees with a DBH of >30 cm (F = 1.69, *p* = 0.046) and more regeneration (F = 7.21, *p* = 0.021) was observed on farmland than in bushland. The average DBH of trees on farmland was 58.78 ± 23.71 cm and seedling density was 15.81 ± 2.04 seedlings per ha (Figure 2B). However, no Tamarind trees in the 1–10 cm, 11–20 cm, and 21–30 cm diameter classes were recorded on farmland (Figure 2B). There was no significant difference in Tamarind tree height (*p* > 0.05) between the two land-use types. The total population status of *T. indica* trees for the two land-use types is shown in Figure 2B.

### 3.2. Tamarind Fruit Yield and Dendrometric Variables

There was a significant difference in Tamarind fruit yield and dendrometric variables between the two land-use types (Table 2). Estimated variables such as number of fruit, fruit yield per tree, DBH, and crown diameter of *Tamarind* were significantly higher for trees on farmland than in bushland (*p* < 0.05) (Table 2). Unlike this, the height and slender ration were greater for the bushland-use type (Table 2). A greater number of fruit were obtained from trees on farmland than in bushland, with a mean value of 4343 on farmland, ranging from 1485 to 8569 fruit per Tamarind tree, and an estimated mean annual yield of 537.05 kg. The mean number of fruit from trees in bushland was 2537, ranging from 1500 to 3500 fruit per tree. The individual fruits weight did not show significant differences (*p* > 0.05) between the two land-use types (Table 2).

**Figure 3:**
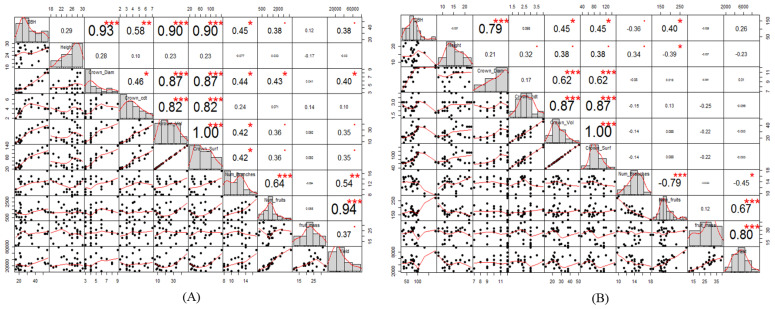
Scatter plot matrices showing correlation coefficients between tree variables and significance levels for Tamarind trees growing in bushland (**A**) and on farmland (**B**) in the Dello Menna district, Southeastern Ethiopia. Abbreviations: DBH, diameter at breast height; Crown_diam, crown diameter; Crown_cdt, crown depth; Crown_vol, crown volume; Crown_surf, crown surface area; Num_branches, number of branches; Num_fruits, number of fruit. On the bottom of the diagonal, bi-variate scatter plots with a fitted line are displayed. On the top of the diagonal, the value of the correlation is shown, plus the significance level of the *p*-values, which are indicated by red asterisks. *p*-values: ***, <0.001; **, <0.01; and *, <0.05.

When correlating variables with Tamarind fruit yield, we found strong correlations between fruit yield and variables such as the number of fruit (0.94) and the number of branches (0.54) (*p* < 0.01; Figure 3A) for trees growing in bushland. A moderate correlation of yield and crown diameter (0.40) was also observed among trees growing in bushland (Figure 3A). The correlation of crown volume, crown surface area, and DBH with Tamarind total yield was weak for trees growing in bushland (Figure 3A). However, there was a strong correlation between fruit yield and variables such as number of fruit (0.80) and fruit yield (0.67) for trees growing on farmland (*p* < 0.001; Figure 3B). A moderate correlation (0.45) was also observed between fruit yield and the number of branches (*p* < 0.05; Figure 3B). Details of correlations between different dendrometric and production variables for Tamarind trees growing on the two land-use types are shown in Figure 3.

### 3.3. Prediction Models to Estimate Fruit Yield of Tamarind

Different dendrometric parameters from the tree size, crown dimensions and tree branches groups were used to fit the model in order to predict the Tamarind fruit yields from the farmland and bushland-use types (Table 3). The dendrometric parameters identified as best from each group were tested in different combinations (16 combinations) to identify the best classification model that included one tree parameter from each group to predict the fruit yield (Table 3).

Four classificatory models (Table 4), three from the farmland-use type and one from the bushland-use type, were selected to predict the fruit yield based on their AIC values (Table 3). The parameters from each group (tree size, crown dimensions, and tree branches) were tested in different combinations to identify the best model that potentially included one tree parameter from each group (Table 4). DBH, crown diameter, crown volume, crown surface area and number of branches were used to predict Tamarind fruit yield from the farmland-use type models, while DBH, crown diameter and number of branches were used for bushland-use type model. In the farmland-use type models, the sign of the coefficients were positive in all of the three fruit yield potential categories (high, low and very high), which indicates that, as the number of parameters increases, total Tamarind fruit yield also increases. Thus, the average fruit yield would increase for every 1 unit increase in the number of tree parameters used in the models for Tamarind trees growing in farmlands of the study area.

## 4. Discussion

To sustainably use and conserve the forest, we need to identify the potential species present and analyze their stand status and production potential, including harvesting level [32]. In this study, a total of 16 woody tree species were associated with the two land-use types studied. Fabaceae was the most dominant family, which is similar to the findings of other studies in the same area [33,34]. This may imply that the environmental conditions in this area are more favorable for species belonging to this family than to other families [34]. The analysis also confirmed that land use impacts woody vegetation, particularly the distribution of *T. indica* in the study area. The density of Tamarind trees was much higher in bushland than in farmland. However, large trees were predominantly growing on farmland, which supports previous findings by Djossa et al. [35], who reported that trees growing on farmland were significantly larger but lower in density than in bushland. This might be because agricultural areas are still farmed in fairly traditional ways and, thus, the overall tree density is low because of regular cropping [36]. However, the condition of these old trees will likely have long-term consequences for the continuity of trees on farmlands, specifically Tamarind trees, and in the long-run, may even lead to population collapse. Thus, the establishment of management plans to create stands that have a more balanced age structure and, thereby, ensure the continuity of the Tamarind population on farmland, is necessary in this study area.

*Dobera glabra* was found in association with *T. indica* on farmlands in the study area. We observed that farmers deliberately retain vital edible fruit trees on their farmlands. Such trees could play a vital role in food security, because their fruit are generally collected for subsistence use by local communities [16,37,38,39]. Furthermore, trees on farms perform important ecological functions, including the provision of soil nutrients [40]. Thus, fruit trees found on farmland in the study area might provide both ecological and social services; however, further empirical studies are needed to verify this assumption. In this regard, the social services included the production of food, fuel, fiber, and other harvestable goods [41]. Also, the cultural services such as recreational, aesthetic, spiritual, and other nonmaterial benefits could be included as the social benefits of farmland trees [42]. Similarly, the ecological services could include the fundamental processes necessary for the production of other services, including soil formation, nutrient cycling and photosynthesis [42].

Although many tropical fruit trees do not have a large world market, their products still have considerable importance in local and national economies and are harvested by rural populations for local consumption and commercialization on a small scale [43]. In this study, the productivity (i.e., the number of fruit per tree and fruit yield/mass) of *T. indica* significantly varied between the two land-use types, with higher levels of productivity recorded for trees growing on farmland than in bushland. Although fruit yield varies seasonally, the number of fresh fruit and the yield per Tamarind tree on farmland was 42% and 64% higher, respectively, than that in bushland. Although the seasonal variation of the yield is usually related with the environmental conditions, the effect may vary with plant species [17]. These results are consistent with the findings of other studies that wild edible trees on farms produce more fruit than unmanaged trees in woodlands [44,45]. Such significant differences in fruit yield under different land-use types might be attributable to differences in environmental variables, such as soil type, landforms, and moisture content [46]. For example, the study on fruit production of *T. indica* across three different ecological regions in Benin indicated that Tamarind trees tend to invest in a small number of very large fruits under wetter conditions and a very large number of small fruits under dryer conditions, indicating the effect of ecological conditions such as temperature and rainfall on the productivity of Tamarind fruits [17]. The level of tree management on farmland has also been reported to significantly influence the degree of competition among trees for available resources such as nutrients [45] and, thus, the productivity of trees in terms of fruit weight and size [44]. Thus, in this study the higher fruit yields from farmland compared with bushland implies that Tamarind trees could benefit from improved management on farms such as hoeing, weeding and reduced competition with other trees [47]. However, although Tamarind trees may tend to produce a large number of high weight fruit under farmland conditions, the majority of the sampled trees produced between 2000 and 5000 fruit per trees. This may indicate that *T. indica* trees growing on farmland have undergone intentional selection owing to local communities removing less-productive trees from farms, suggesting that fruit yields could be further improved through deliberate selection of superior yielding trees from farmland types in the study area. Thus, further surveys of trees on farmland should be carried out to identify superior germplasm that could be used to play a vital role in enhancing fruit production as part of the domestication process of this species.

The relationship between fruit yield per tree and tree dendrometric parameters was expressed by multinomial classification model. The model with grouped dendrometric parameter predictor such as tree size, crown structure and tree branches yielded reliable equations with the lowest AIC values. In order to increase the practical usefulness of the model and to avoid large errors in the estimation of Tamarind fruit yield, models with one dendrometric parameter predictor from each group were chosen. The identified model allowed an accurate fruit yield estimation obtained from the farmland- and bushland-use types of the study area. In this regard, the models holding the combination of DBH, crown diameter, crown volume, crown surface area and number of branches were used to predict Tamarind fruit production from the farmland-use type, while the parameters of the DBH, crown diameter and number of branches were used to develop models for bushland-use type. Similar types of studies have also concluded that the chosen tree parameters best predicted the fruit yield of *Vitex* trees under different land-use conditions [47], whereas other studies have suggested that DBH measurements better estimate fruit yields of *Baobab* [48] and *Vitex* trees [27] using a linear regression models. However, the tree size, crown structure and number of branches of Tamarind trees are affected by environmental and geographical factors such as rainfall, temperature, latitude and longitude, and soil conditions [49,50], which directly affect the accuracy of the models when used for fruit estimation in other places than the study area. Thus, the use of site-specific model may be recommendable for more accurate fruit yield estimations of Tamarind trees. On the other hand, the tree slenderness often serves as an index of tree stability, or the resistance to wind throw [51], although the likelihood of wind throw of a tree may be also influenced by different interacting factors. In this study, the result indicated that the tree slenderness value of the bushland-use type is higher than that of the farmland, indicating that the higher slenderness occurs for the trees with higher height length and smaller diameter distributions. The smaller slenderness is usually indicating a higher resistance to wind throws. Thus, tree treatments, such as producing long-crowned trees, and maintaining appropriate stand density through spacing, thinning, or gradually harvesting over story trees, can be helpful in reducing the risk of wind throw of the farmland-use Tamarind trees in the study area. In this study, the correlation of dendrometric parameters suggested that under enhanced management, Tamarind trees in both land-use types could achieve better yields and structure. However, since *T. indica* can be grown in a diverse dryland area of Ethiopia, a comprehensive study considering management and germplasm selection is required to validate the equation developed in this study.

## 5. Conclusions

This study highlighted the population status of *T. indica* trees under two types of land-use in Dello Menna, Southeastern Ethiopia. We have also provided useful preliminary information on the variation in fruit yield in Tamarind of the two land-use types. Overall, there were a higher number of seedlings and large sized Tamarind trees per hectare on farmland than in bushland, indicating that matured trees are well preserved on farmlands. However, we could not find tree size classes between seedling and mature trees on farmland, whereas well-structured tree size classes were observed in bushland. This situation is likely to have long-term consequences for the population continuity of Tamarind trees on farmland in the long run. Thus, management plans to develop meaningful stand structures on farmland need to be established to ensure the continuity of the Tamarind population in the studied area, either by planting new Tamarind seedlings in specific plots sites or by conserving some of the existing seedlings during the preparation of the soils for new cultivation. Although higher Tamarind fruit yields were obtained from trees growing on farmland than in bushland in the Dello Menna district, the majority of farmland trees produced <5000 fruit per tree.

Classificatory models revealed that, among the tree size variables, DBH was more suitable to predict the fruits yield category than the height of the trees. Similar results were observed for crown related variables such as crown depth, crown volume and crown surface in the farmlands, whereas crown diameter provide the best results in the bushlands. Finally, the number of branches was always significantly related, as we expected, with the yield classification. Thus, the obtained models indicated that the fruit yields of Tamarind trees could be improved through management under different tree size, crown structure and tree branch category. Furthermore, the yield improvement can be done through deliberate selection of higher fruit yielder trees, taking into consideration that Tamarind could grow in more diverse dryland areas in Ethiopia. These strategies might also lead to the development of a sustainable fruit supply in rural areas for commercialization or subsistence use. The findings from this study provide baseline information that could be used to promote the conservation and sustainable uses of valuable wild edible tree species such as Tamarind under different land-use types in Ethiopia. The application of this baseline information is immense and could also assist other developing countries with valuable wild edible trees that are facing similar issues due to land-use change. However, as the present study was based on single-year data, several years of monitoring are needed to precisely model the yield production, taking into consideration the environmental variables such as soil, rainfall, temperature, together with tree genetic variation and inter-annual variation in fruit yield in order to understand the Tamarind productivity better in the study area.

## Figures and Tables

**Table 1:** Densities of woody tree species growing on farmland and bushland in the study area in Dello Menna, Southeastern Ethiopia.

Species	Family	Density (Trees ha^−1^)	
		Farmland	Bushland
*Acacia etbaica* Schweinf.	Fabaceae	3.95 ± 0.012	2.00 ± 0.001
*Acacia mellifera* (M. Vahl) Bentham	Fabaceae	–	1.55 ± 0.010
*Acacia senegal* Willd.	Fabaceae	3.95 ± 0.012	10.82 ± 0.024
*Acacia tortilis* (Forssk.) Hayne	Fabaceae	98.79 ± 0.095	26.27 ± 0.024
*Carissa edulis* (Edgew.) Benth.	Apocynaceae	–	1.54 ± 0.007
*Combretum molle* R.Br. ex G.Don	Combretaceae	–	2.00 ± 0.010
*Commiphora baluensis* Jacq.	Combretaceae	–	26.27 ± 0.07
*Dobera glabra* (Forssk.) Juss. ex Poir.	Salvadoraceae	15.81 ± 0.035	7.73 ± 0.011
*Euclea racemosa* L.	Ebenaceae	–	1.55 ± 0.007
*Grewia bicolor* Juss.	Malvaceae	–	1.55 ± 0.001
*Grewia villosa* Willd.	Malvaceae	–	1.54 ± 0.007
*Lannea schimperi* (A. Rich.) Engl.	Anacardiaceae	–	3.09 ± 0.008
*Ochna inermis* (Forssk.) Schweinf	Ochnaceae	–	1.55 ± 0.005
*Tamarindus indica* L.	Fabaceae	23.71 ± 0.050	35.54 ± 0.049
*Terminalia polycarpa* Engl. & Diels	Combretaceae	–	1.56 ± 0.001

Note: the number of stems per hectare includes both mature trees and regenerated seedlings. Values shown are means ± standard deviation.

**Table 2:** Dendrometric variables (± standard deviation) of *Tamarindus indica* trees for two land-use types in the study area, Dello Menna district, Southeastern Ethiopia.

Tree Characteristic	Land-Use		*F*-Value	*p*-Value
	Farmland	Bushland		
Diameter (cm)	52.22 ± 8.43	30.56 ± 13.31	51.08	0.0000
Height (m)	9.78 ± 1.78	16.30 ± 5.13	38.82	0.0002
Tree slender ration	0.23±0.12	0.95 ± 0.36	93.28	0.0000
Crown diameter (m)	10.26 ± 1.48	4.18 ± 0.93	4.02	0.0000
Number of fruit per tree	4343 ± 1996	2537.04 ± 615.13	20.17	0.0001
Individual fruits weight (g)	26.02 ± 1.19	22.74 ± 6.21	1.57	0.2158
Fruit yield (kg) per tree	537.05 ± 255.16	172.81 ± 62.82	51.78	0.0000

**Table 3:** Combination of results fitted multinomial classificatory models to fruit yield of *Tamarindus indica* trees for two land-use types in the study area, the Dello Menna district, Southeastern Ethiopia.

Land-Use Type	Tree Size		Crown Dimensions				Tree Branches	AIC
	DBH	Height	Crown Diameter	Crown Depth	Cvol	CSA	NB	
Farmland	1		1				1	60.30
Farmland	1			1			1	57.18
Farmland	1				1		1	57.17
Farmland	1					1	1	57.17
Farmland		1	1				1	59.56
Farmland		1		1			1	63.18
Farmland		1			1		1	62.45
Farmland		1				1	1	62.29
Bushland	1		1				1	87.74
Bushland	1			1			1	88.80
Bushland	1				1		1	88.66
Bushland	1					1	1	88.62
Bushland		1	1				1	88.73
Bushland		1		1			1	88.98
Bushland		1			1		1	89.19
Bushland		1				1	1	89.19

Note: DBH: diameter at breast height, Cvol: crown volume; CSA: crown surface area, NB: number of branches and AIC: Akaike Information Criterion.

**Table 4:** *Tamarindus indica* fruit yield production (kg ha^−1^) multinomial classificatory models based on yield category for the two land-use types in the study area, Dello Menna district, Southeastern Ethiopia.

Model	Land Uses	Yield Category	Intercept	Tree Size		Crown Dimensions				Nbr
				DBH	H	Cdr	Cdt	Cvol	CSA	
1	Farmland	High	41.6988	−0.4059	-	-	3.4148	-	-	−0.9906
		low	31.0819	−0.4160	-	-	4.5007	-	-	−0.4235
		Very high	0.7668	0.5824	-	-	−2.5525	-	-	−5.5457
2	Farmland	High	39.2695	−0.3716	-	-	-	0.2177	-	−0.8997
		low	30.0463	−0.4041	-	-	-	0.3275	-	−0.3404
		Very high	1.8206	1.2428	-	-	-	−2.7251	-	−4.6551
3	Farmland	High	38.8419	−0.3674	-	-	-	-	0.0714	−0.8877
		low	29.6071	−0.4000	-	-	-	-	0.1080	−0.3276
		Very high	1.2811	0.8070	-	-	-	-	−0.2852	−5.1273
4	Bushland	High	−2.1487	0.0053	-	0.3524	-	-	-	−0.0777
		low	2.4140	0.1327	-	−0.9756	-	-	-	−0.1184
		Very high	−3.0593	0.0837	-	−0.6959	-	-	-	0.2844

Note: DBH: Diameter at breast height, H: height, Cdr: crown diameter, Cdt: crown depth, Cvol: crown volume, CSA: crown surface area and Nbr: number of branches.

## Data Availability

Not applicable.
